# Predictive risk factors for lymph node metastasis in patients with resected non-small cell lung cancer: a case control study

**DOI:** 10.1186/s13019-019-0831-0

**Published:** 2019-01-16

**Authors:** Yusef Moulla, Tanja Gradistanac, Christian Wittekind, Uwe Eichfeld, Ines Gockel, Arne Dietrich

**Affiliations:** 1 Department of Visceral, Transplant, Thoracic and Vascular Surgery, University Hospital of Leipzig, Liebigstraße 20, 04103 Leipzig, Germany; 2 Institute of Pathology, University Hospital of Leipzig, Liebigstraße 20, 04103 Leipzig, Germany

**Keywords:** Non-small cell lung cancer (NSCLC), Lymph node metastasis, Lymphatic vessel invasion

## Abstract

**Background:**

Estimation of lymph node status is essential in order to determine precise therapy for patients with non-small cell lung cancer (NSCLC). Furthermore, lymph node involvement is a very powerful prognostic factor in these patients. In this analysis, we aim to evaluate the predictive factors for lymph node metastasis in NSCLC-patients.

**Methods:**

In a prospectively-established database, we analyzed all data of patients with NSCLC, who underwent oncological surgical resections from 01/2007 to 12/2016, retrospectively. The correlation between clinicopathological parameters and lymph node metastasis was investigated by using univariate and binary logistic regression analysis.

**Results:**

In this study, we operated on 204 consecutive patients, 142 men (71.7%) and 56 women (28.3%). Lymph node metastases were detected in 38.2% (78/204). Preoperatively, central tumor localization (OR = 2.6, 95% CI = 1.3–5.1, *P* = 0.005) and tumor size > 3 cm (OR = 2.5, 95% CI = 1.3–4.4, *P* = 0.005) were found to be significant predictive factors for lymph node metastasis. Postoperatively, multivariate analysis showed that intratumoral lymph vessel invasion (L1-status) (OR = 17.3, 95% CI = 5.1–58.4, *P* <  0.001) along with the central tumor localization (OR = 2.8, 95% CI = 1.4–5.8, *P* = 0.004) were significantly associated with lymph node metastasis. In small size tumors (≤3 cm), two predictive factors for lymph node metastasis were found: central tumor localization (OR = 19.4, 95% = 2.1–186.4, *P* = 0.01) and L1-status (OR = 43.9, 95% CI = 3.6–529.4, *P* = 0.003).

**Conclusions:**

A precise pre- and intraoperative assessment of the lymph node status is essential in patients with larger sized tumors and central localization. Furthermore, L1-status is a highly significant risk factor for lymph node metastasis in NSCLC-Patients. Therefore, an adjuvant therapy in patients with L1-status and pNX category should be considered.

## Background

Lung cancer is the leading cause of cancer-related death worldwide [1]. The main histological type of lung cancer is non-small lung cancer (NSCLC) (80–85%). A correctly defined stage of this entity is crucial for therapy planning and estimating of prognosis. The nodal status is considered as one of the most important prognostic factors in NSCLC-patients and, thus, essential in determining the perioperative therapy [2]. Several techniques have been described to detect the clinical N-category, such as radiologic imaging, endoscopic and surgical techniques. The indication to use invasive investigations procedures is always thoroughly discussed to avoid their complications and unnecessary costs. In addition, the extent of intraoperative lymphadenectomy to achieve an accurate staging of NSCLC is by itself a controversial topic. Systematic nodal dissection (SND) with removal of all ipsilateral hilar and mediastinal lymphatic tissue is recommended in oncological lung resection [3]. Other limited node dissection techniques, such as systematic sampling, are considered as effective as SND for accurate staging [4, 5]. As mentioned, it is hard to find the most cost-effective and highest-quality investigation or procedure to accurately determine the stage of the specific lung cancer. In this study, we aimed to identify predictive factors for lymph node metastasis in patients with NSCLC undergoing surgery.

## Materials and methods

### Patients

All surgically treated NSCLC-patients in curative intent at the University Hospital of Leipzig from 01/2007 to 12/2016 were documented in a prospective database and retrospectively evaluated. Patient’s TNM-classifications of the former 6th &7th edition were transferred into the definitions of 8th edition of American Joint Committee on Cancer (AJCC) by our pathologists in order to gain comparable tumor stages [6]. Only patients, who underwent oncological surgical resections with SND, were analyzed. Exclusion criteria were: (i) Any neoadjuvant therapy or (ii) patients with limited/palliative resections and (iii) those with carcinoid tumors. Clinical staging included mainly Blood tests, Thorax-CT, PET/CT, eventually Brain MRI (symptomatic patients) and Bronchoscopy. Invasive mediastinal staging such as Endobronchial Ultrasound (EBUS) was only done by patients with cN2, cN3 according to the radiological findings. SND was performed according to the technique as described by Graham et al. [7]. The mediastinal lymph nodes were dissected systematically within anatomical landmarks und labelled with respect to the Mountain & Dresler map (MD-ATS map) [8] till 2010, and thereafter according to the map of the International Association for Study of Lung Cancer IASLC [9]. The hilar station (No. 10) was dissected before lung resection. Other regional pulmonary lymph nodes (stations No. 11–14) were dissected simultaneously within the resected lung. The dissection of the mediastinal lymph nodes was standardly performed after the resection. For right sided tumors, the lymph nodes stations No. 2, 3 and 4 were removed after opening the mediastinal pleura above the azygos vein. Lymph nodes of the lower mediastinum in stations No. 7, 8 and 9 were dissected after mobilization of the lower lobe. For left-sided tumors, the lymph node dissection was commenced at the aortic arch (stations No. 5 and 6) after locating and conserving the vagal nerve and its branch, the recurrent laryngeal nerve. Then, we proceeded to the dissection to the upper mediastinum (tracheobronchial lymph node stations 2&4), which located medial to the subaortic lymph nodes. These lymph nodes could be dissected after mobilization the aortic arch anteriorly (Ligament of Botalli could be divided) and by giving extreme care of the left recurrent laryngeal nerve and left vagal nerve. The Lymph node stations in lower mediastinum (stations No. 7, 8 and 9) were dissected similarly to the right side. The tumor localization was defined as central, if the lesion had been visible endoscopically during preoperative bronchoscopy. The nodal status was worked-up and classified by the pathologist after surgery.

In this study, factors such as age, gender, histological tumor type, tumor site, size, localization and lobe distribution were examined as to their effect on lymph node metastasis in patients with resected NSCLC. Furthermore, histological and morphological parameters, such as pT- category, R-status, lymphatic vessel invasion status (L-status) and venous invasion status (V-status), histological tumor type and grading (G) were investigated postoperatively in terms of their association of lymph node metastasis. We also analyzed the predictive factors for lymph node metastasis in particular in patients with pT1-category, as this cohort is described to develop limited lymph node metastasis only.

### Statistical analysis

To identify potential predictors for pulmonary and mediastinal lymph node metastasis, we used univariate analyses and multivariate binary logistic regression analyses.

In the univariate analysis, Pearson’s Chi-square and, if necessary, Fisher exact-test, were used to investigate the correlation of lymph nodes metastasis with categorical variables, and independent sample t-test as well as Mann- Whitney-U-test, to evaluate this association with continuous/discrete variables.

Patient’s variables with *P*-value less than 0.2 were entered stepwise into a multivariate binary logistic regression model, which formed the basis of two prediction models. All factors that reached a significance level of .05 remained in the two final models.

We developed two models for the prediction of lymph node metastasis: The first one for the preoperative phase according to the radiologic und endoscopic findings, and the second one after the surgical resection according to the preoperative findings and the postoperative histopathological reports. We used SPSS (v. 20.0) for Windows 10 for these statistical analyses. All tests were 2-sided.

## Results

### Patient^’^s characteristics

Between January 2007 and December 2016, a total of 204 patients with NSCLC fulfilling the inclusion criteria underwent oncologic resection with SND. They were 142 males (71.7%) and 56 females (28.3%). The gender ratio (men to women) was [2.5:1]. The median age was 68 years, range [33 to 85]. Right sided tumors were observed in 61.8% (126/204) and left sided in 38.2% (78/204). The right upper lobe was the most frequent localization of tumors with 38.2% (78/204).

The main procedure in our patient cohort was lobectomy (77.9%, *n* = 159). Extended resection including bronchoplastic procedures and/or vascular involvement were performed in 17 cases (8.4%) of all resections. SND was carried out in all patients in a standardzed manner. Histologically, the ratio between adenocarcinoma and squamous cell carcinoma (SCC) was 1.5 to 1 (Table 1). The mean size of the tumors was 3.6 ± 2.2 cm. The median number of dissected lymph nodes was 25, range [6 to 69]. Tumor positive lymph nodes (pN+) were found in 38.2% (78/204) postoperatively. Pulmonary lymph node metastases (pN1+, pN2-) were depicted in 16.2% (33/204). 17.2% (35/204) of all patients had pulmonary and mediastinal lymph node metastases (pN1+, pN2+). A skip-phenomenon (pN1-, pN2+) was observed in 4.9% (10/204) of patients. Mediastinal lymph node station 4 was the most positive station in all patients with pN2+ (20/45, 44.4%). pR0-status was confirmed in 93.1% of all cases (189/204). The postoperative TNM-classification (pTNM) is shown in (Table 1).

**Table 1:** Main characteristics of the patients, pTNM-Classification 2017, 8th Edition

Characteristics	Number of Patients (n)	Percentage
Tumor-localization		
central	53	26.2%
peripheral	149	73.8%
Histology		
adenocarcinoma	117	57.4%
SCC^a^	75	36.8%
others	12	5.9%
Grading		
In situ	2	1%
G1	25	12.4%
G2	95	47.0%
G3	68	33.3%
G4	12	5.9%
pT-category		
pTis	2	1%
pT1	90	45.1%
pT1a	10	4.9%
pT1b	37	18.1%
pT1c	43	21.1%
pT2	44	26.5
pT2a	39	19.1%
pT2b	15	7.4%
pT3	35	17.2%
pT4	23	11.3
pN-category		
pN0	126	61.8%
pN1	33	16.2%
pN2	45	22%
pM-category		
pM0	193	94.5%
pM1a	5	2.5%
pM1b	5	2.5%
pM1c	1	0.5%
pL-Status		
pL0	57	28.1%
pL1	146	71.9%
pV-Status		
pV0	165	81.3%
pV1	38	18.7%

^a^*SCC* Squamous cell carcinoma

### Univariate analysis of predictive factors for lymph node metastasis in resected NSCLCs

Among the investigated parameters, univariate analysis identified the following variables as significant predictors for pulmonary lymph node metastasis (pN1+): Increased tumor size, central tumor localization, pT-category, R-classification, L- and V-status as well as a higher grading (*P* <  0.05). Other potential parameters, such as age, histological tumor type and further clinicopathological features showed no statistically significant correlation with pulmonary lymph node metastasis (pN1+) (Table 2). The same predictors proved significant for the existence (pulmonary and/or mediastinal) of any lymph node metastasis (pN+) (Table 3). Furthermore, increased tumor size, central tumor localization, L- and V-status as well higher grading were also identified as significant predictors for mediastinal lymph node metastasis (pN2+) (*P* < 0.05) (Table 2).

**Table 2:** Univariate Analysis of positive pulmonary and mediastinal lymph nodes (pN1+, pN2+)

Variables	pN1- (%)	pN1+ (%)	*P*-Value	pN2- (%)	pN2+ (%)	*P*-Value
Sex			0.148			0.627
male	90 (63.4)	52 (36.6)		112 (78.9)	30 (21.1)	
female	46 (74.2)	16 (25.8)		47 (75.8)	15 (24.2)	
Age			0.298			0.237
≤ 68 years	68 (63)	40 (37)		88 (81.5)	20 (81.5)	
> 68 years	68 (70.8)	28 (29.2)		71 (74.0)	25 (21.2)	
Tumor site			0.067			0.783
right	90 (71.4)	36 (28.6)		99 (78.6)	27 (21.4)	
left	46 (59)	32 (41)		60 (76.9)	18 (23.1)	
Tumor localization			< 0.001			0.02
central	25 (47.2)	28 (52.8)		36 (66.7)	18 (33.3)	
peripheral	111 (74.5)	38 (25.5)		122 (81.9)	27 (18.1)	
Tumor size			0.011			0.042
≤ 3 cm	77 (75.5)	25 (24.5)		86 (84.3)	16 (15.7)	
> 3 cm	59 (57.8)	43 (42.8)		73 (71.6)	29 (28.4)	
Lobe distribution			0.918			0.385
UL^a^	85 (66.4)	43 (33.6)		97 (75.8)	31 (24.2)	
Non-UL	51 (67.1)	25 (32.9)		62 (81.6)	14 (18.4)	
pT-category			0.015			0.081
pT1	70 (77.8)	20 (22.2)		77 (85.6)	13 (14.4)	
pT2	33 (61.1)	21 (38.9)		40 (74.1)	14 (25.9)	
pT3	18 (51.4)	17 (48.6)		23 (65.7)	12 (34.3)	
pT4	13 (56.5)	10 (43.5)		17 (73.9)	6 (26.1)	
pR-classification			0.017			0.088
R0	130 (68.8)	59 (31.2)		150 (79.4)	39 (20.6)	
R1–2	5 (35.7)	9 (64.3)		8 (57.1)	6 (42.9)	
pL-status			< 0.001			< 0.001
pL0	55 (96.5)	2 (3.5)		56 (98.2)	1 (1.8)	
pL1	80 (54.8)	66 (45.2)		102 (69.9)	44 (30.1)	
pV-status			0.006			0.004
pV0	117 (70.9)	48 (29.1)		135 (81.1)	30 (18.2)	
pV	18 (47.4)	20 (52.6)		23 (60.5)	15 (39.5)	
Pn-Status			0.088			0.34
Pn0	131 (67.9)	62 (32.1)		149 (77.2)	44 (22.8)	
Pn1	4 (40.0)	6 (60.0)		9 (90.0)	1 (10.0)	
Histological tumor type			0.214			0.646
Adeno-Ca.^b^	84 (71.8)	33 (28.2)		94 (80.3)	23 (19.7)	
				56 (74.4)	19 (25.6)	
SCC^c^	45 (60.0)	30 (40.0)		9 (75.0)	3 (25.0)	
others	7 (58.3)	5 (41.7)				
Grading			0.016			0.001
G1	25 (89.3)	3 (10.7)		27 (96.4)	1 (3.6)	
G2	64 (67.4)	31 (32.6)		79 (83.2)	16 (16.8)	
G3	39 (57.4)	29 (42.6)		43 (63.2)	25 (36.8)	
G4	7 (58.3)	5 (41.7)		9 (75.0)	3 (25.0)	

^a^*UL* Upper lobe,^b^ *Adeno-Ca.* Adenocarcinoma , ^c^*SCC* Squamous cell carcinoma

**Table 3:** Univariate Analysis of positive lymph nodes (pN0, pN+)

Variables	pN0 (%)	pN+ (%)	*P*-Value
Sex			0.397
male	85 (59.9)	57 (40.1)	
female	41 (66.1)	21 (33.9)	
Age			0.314
≤ 68 years	63 (58.3)	45 (41.7)	
> 68 years	63 (65.6)	33 (34.4)	
Tumor site			0.067
Right	84 (66.7)	42 (33.3)	
Left	42 (53.8)	36 (46.2)	
Tumor localization			< 0.001
central	22 (41.5)	31 (58.5)	
peripheral	104 (69.8)	45 (30.2)	
Tumor size			< 0.001
≤ 3 cm	76 (74.5)	26 (25.5)	
> 3 cm	50 (49)	52 (51)	
Lobe distribution			0.834
UL ^a^	78 (60.9)	50 (39.1)	
Non-UL	40 (62.5)	24 (37.5)	
Number of dissected LN ^b^			0.005
pT-category			0.001
pT1	69 (76.7)	21 (23.3)	
pT2	27 (50.9)	26 (49.1)	
pT3	17 (47.2)	19 (52.8)	
pT4	11 (47.8)	12 (52.2)	
pR-classification			0.048
pR0	120 (96.0)	5 (4.0)	
pR1–2	69 (88.5)	9 (11.5)	
pL-status			< 0.001
pL0	54 (94.7)	3 (5.3)	
pL1	71 (48.6)	75 (51.4)	
pV-status			0.001
pV0	111 (67.3)	54 (32.7)	
pV1	14 (36.8)	24 (63.2)	
Pn-status			0.188
Pn0	121 (96.8)	4 (3.2)	
Pn1	72 (92.3)	6 (7.7)	
Histological tumor type			0.141
Adeno-Ca.^b^	79 (67.5)	38 (32.5)	
SCC^c^	41 (54.7)	34 (45.3)	
others	6 (50.0)	6 (50.0)	
Grading			0.005
G1	22 (88.0)	3 (12.0)	
G2	60 (63.2)	35 (36.8)	
G3	34 (50.0)	34 (50.0)	
G4	6 (50.0)	6 (50.0)	

^a^*UL* Upper lobe, ^b^*Adeno-Ca.* Adenocarcinoma, ^c^ *SCC* Squamous cell carcinoma

### Multivariate analysis of predictive factors for lymph node metastasis in resected NSCLCs

The multivariate analysis revealed, according to the preoperative findings, the following significant predictive factors for pulmonary (pN1+) and mediastinal (pN2+) lymph node metastasis: Central tumor localization and increased tumor size (Table 4). Furthermore, this multivariate analysis identified tumor size > 3 cm and central tumor localization as significant factors for any lymph node metastasis (pN+) (Table 4).

**Table 4:** Multivariate analysis of predictive factors for lymph node metastasis (preoperative model)

Predictive factors	Pulmonary LN ^a^ metastasis (pN1+)	Mediastinal LN ^a^ metastasis (pN2+)	Any LN ^a^ metastasis (pN+) ^e^
Central localization	*P* = 0.002 ^b^ OR = 2.9 ^c^ CI = 1.5–5.6 ^d^	*P* = 0.06 OR2.0 CI = 0.9–4.1	*P* = 0.005 OR = 2.6 CI = 1.3–5.1
Increased tumor size	*P* = 0.043 OR = 1.2 CI = 1.0–1.3	*P* = 0.040 OR = 1.2 CI = 1.0–1.4	*P* = 0.005 OR = 2.5 CI = 1.3–4.4

^a^*LN* Lymph node, ^b^*P* *P*-Value, ^c^*OR* Odds ratio, CI 95%, ^d^*CI* Confidence interval, ^e^ Patients were divided in two groups according to tumor size (> 3 cm and ≤ 3 cm)

According to the preoperative and postoperative findings, multivariate analysis revealed two significant predictive factors for lymph node metastasis (pN+): central tumor localization (OR = 2.8, 95% CI = 1.4–5.8, *P* = 0.004) and L1-status (OR = 17.3, 95% CI = 5.1–58.4, *P* < 0.001). Other factors, such as age, histologic tumor type and grading, tumor site, tumor lobe distribution, R-status, V- and Pn-status, did not prove predictive for lymph node metastasis.

### Lymph node metastasis in pT1 NSCLCs

In this study, 90 patients (45.1%) had a pT1 carcinoma. The median age was 67 years, range [45 to 84]. There were 63.3% males (57/90) and 36.7% females (33/90). Gender ratio was 1.7:1 (men to women). Right sided tumors were found in 66.7% (60/90). A central tumor localization was detected in 10 patients (11.1%). The median tumor size was 2 cm, range [0.4 to 3 cm], whereas 52.2% of the patients displayed tumor size ≤2 cm (*n* = 47/90). Pathological types were adenocarcinomas (*n* = 64, 71.1%), suqamous cell carcinomas (SCC) (*n* = 21, 23.3%), and others (*n* = 5, 5.6%). Grading G3 and G4 were detected in 28 patients with pT1 carcinomas (31.5%). The median number of dissected lymph nodes was *n* = 22, range [6 to 67]. Lymph node metastases (pN+) were found in 19 patients (21.1%), pulmonary lymph node metastases (pN1+) in 15 patients (16.6%), and mediastinal lymph node metastases (pN2+) in 12 patients (13.3%). L1-status was found in 48 patients of this group (53.3%).

### Univariate and multivariate analysis of predictive factors for lymph node metastasis in pT1 NSCLCs

The univariate analysis identified the following predictive factors as significant for lymph node metastasis (pN+): Central tumor localization (*P* = 0.005), L-status (*P* < 0.001), V-status (*P* = 0.019) and pathological grading (*P* = 0.009). Other clinicopathological parameters, such as age, gender, tumor site, tumor lobe distribution, histological tumor type and the number of dissected lymph nodes did not correlate with a *P*-value> 0.2. Tumor size was also not significant but had a *P*-value< 0.2 (*P* = 0.1).

**Fig. 1:**
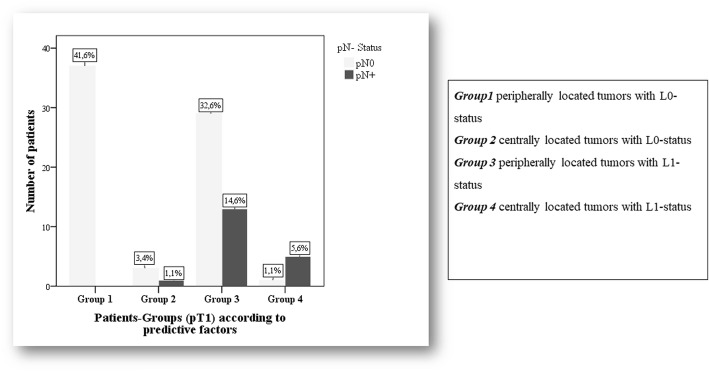
pN-category in patients with pT1-tumors according to predictive risk factors

All parameters with a significance level < 0.2 were included in the multivariate analysis to determine the predictive factors for lymph node metastasis. This analysis revealed only two significant risk factors for lymph node metastasis: central tumor localization (OR = 19.4, 95% CI = 2.1–186.4, *P* = 0.01) and L1 status (OR = 43.9, 95% CI = 3.6–529.4, *P* = 0.003) (Fig. 1).

## Discussion

We identified increased tumor size and central tumor localization as significant predictive factors for pulmonary and mediastinal lymph node metastasis in resected NSCLCs, according to the preoperative radiologic und endoscopic findings. Furthermore, a tumor size > 3 cm was in addition to the central tumor localization a significant factor for any lymph node metastasis (pN+).

In an analysis of 566 patients (175 patients with pN2+), Shafazand et al. [10] found tumor size > 3.6 cm and central tumor localization as significant predictive factors for lymph node metastasis. This study also identified age of < 65 years and histological tumor type (adenocarcinoma or large cell) as predictive factors for lymph node metastasis. Another retrospective analysis of 379 patients (68 with pN2) by Suzuki et al. [11] demonstrated that tumor size > 2.0 cm, high serum carcinomembryonic antigen (CEA) level and the histopathological tumor type (adenocarcinoma) were predictive factors for mediastinal lymph node metastasis. In our study, we could not identify age or the pathological entity as predictive factors for any lymph node metastasis. Unfortunately, CEA level was not available in our database and, thus, not included in the current analysis. However, this serum tumor marker has been described in previous studies as a predictive factor for advanced disease and worse prognosis [12]. Graham et al. [7] detected younger age, increased tumor diameter, left lower lobe origin and bronchial origin as significant predictive factors for lymph node metastasis in resected NSCLCs. In our study, tumor localization in lower left lobe (LLL) proved without any influence on lymph node metastasis (*P* = 0.448) and, thus, we were unable to replicate these data found by Graham et al.

Accurate mediastinal staging in patients with operable NSCLCs preoperatively and postoperatively is very essential to guide the treatment and to determine the extent of disease and consequently the patient^’^s prognosis. This staging can be performed through radiologic, endoscopic and surgical techniques. Previous meta-analyses have already shown that the use of thorax-CT in assessing the cN-category has a sensitivity of 60–83%, a specificity of 77–82% and a negative predictive value of 80–85% [13, 14].

Another meta-analysis compared PET/CT and diffusion-weighted magnetic resonance imaging (DW-MRI) technique in detecting the mediastinal lymph node metastasis preoperatively [15]. Sensitivity was low at 65% for PET/CT and 72% for DWI, while specificity was high (93% vs. 97%). Overall, the assessment of lymph node micrometastasis by radiologic investigations is not reliable. Endoscopic procedures, such as Endobronchial Ultrasound (EBUS) with Transbronchial Needle Aspiration (TBNA), provide more accurate information about the mediastinal staging, with a sensitivity of 83–94% [16]. Surgical techniques, such as video-assisted mediastinoscopy (VAM) (accuracy 88–99%) and video-assisted mediastinal lymphadenectomy (VAMLA) (accuracy 100%) have the highest accuracy with respect to the preoperative mediastinal staging. However, these techniques have a morbidity rate of 3.5% [17]. Intraoperative evaluation of nodal status at the hilar and mediastinal stations is known to be a very important tool for accurate staging [18]. As mentioned above, the pathological examination of dissected lymph nodes revealed the most precise assessment of nodal status in patients with NSCLCs.

The necessary extent of lymph node dissection in the treatment of NSCLC remains controversial [18, 19]. Some authors have reported performing SND in all oncological resections of NSCLC [20, 21]. Others have recommended systematic mediastinal lymph node sampling (MLNS) or lobe-specific node dissection as good options in operable NSCLCs, in order to estimate the lymph node status and to reduce the perioperative morbidity and mortality [4, 22]. While the effectiveness of SND to improve local tumor control and to achieve accurate staging has already been established, the benefit of SND regarding prognosis is yet to be proven [3]. Keller et al. [23] demonstrated improved survival and detection of more positive N2 lymph nodes (upstaging) through SND than with MLNS. Furthermore, Shapiro et al. [24] found similar survival rates in a lobe specific lymph node dissection group as compared to the SND group, following either lobectomy via thoracoscopy (VATS) or thoracotomy in clinically negative N2 patients. Minimally invasive surgery has widely been established as an appropriate surgical choice for early stage NSCLC patients, but the extent and feasibility of lymph node dissection during minimally invasive thoracic surgery in patients with clinically negative N2 disease still remains a controversial topic. Toker et al. [25] proved that minimally invasive thoracic surgery, including robotic assisted thoracic surgery, can harvest a similar number of lymph nodes as compared to open surgery. Other authors, such as Merritt et al. [26], found a significantly higher mean number of dissected lymph nodes by open lobectomy than by VATS-lobectomy. In addition, the overall pathological upstaging of the pN-status was significantly higher in the open lobectomy group [26].

**Fig. 2:**
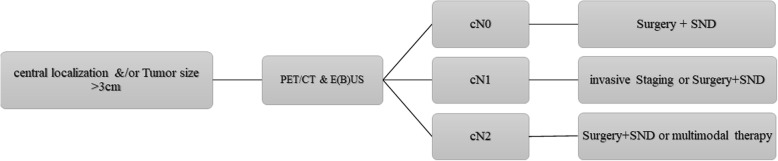
Suggested algorithm to achieve a precise assessment of lymph node metastasis in NSCLC patients with risk factors

Considering the above-mentioned studies and our own results, we would suggest that a more precise preoperative or intraoperative assessment of all lymph node stations in patients with large size tumors and/or with central tumor localization is mandatory. The surgical technique used should not prevent a precise assessment of hilar and mediastinal lymph nodes. We therefore insist on the following recommendation of the European Society of Thoracic Surgery (ESTS) guidelines, which state, that the most sensitive procedures (endoscopic and surgical), even when invasive, should be performed to achieve an accurate mediastinal staging in patients with centrally located and/or large size (> 3 cm) tumors [17]. Following our results from this study and in consideration of those from others, [17, 18] we started applying the algorithm shown in Fig. 2 in our department, in order to achieve a precise preoperative and intraoperative assessment of lymph node metastasis in patients with high risk factors.

In this study, we identified the rate of lymph node metastasis in patients with small size tumors (pT1 carcinomas) to be approximately 20%, which is similar to other international studies [27]. The necessary extent of surgical resection, preoperative mediastinal staging and intraoperative lymph node dissection in small size tumors (≤3 cm) is another controversial topic. L1-status and central localization were found to be significant predictive factors for the existence of lymph node metastases. However, tumor localization can be detected easily preoperatively. This information can help us in advance to determine the appropriate procedure and the extent of surgical resection and lymphadenectomy. Many studies have recommended a limited surgical resection (e.g. segmentectomy) to be oncologically adequate in patients with small size tumors (T1 carcinomas) [28, 29]. However, the precise assessment of pulmonary lymph nodes in limited resections is still a field of debate in the surgical community. Therefore, limited surgical resections in patients with centrally located tumors are not recommended so far, as these do not allow for efficient dissection of pulmonary and hilar lymph nodes. The need for invasive mediastinal staging (endoscopic −/+ surgical techniques) in patients with centrally located small size tumors should be evaluated in accordance with the results of this study.

The other parameter (L-status) is rarely found in the preoperative pathological reports. The presence of L1-status is associated with a higher rate of local recurrence and a worse overall survival in early lung cancer [30]. Higgins et al. [31] described L1-status as a risk factor for regional lymph node involvement as well as a poor prognostic factor in NSCLC-patients. Such informations can be useful in determining the recommended treatment in patients who have undergone limited resection without complete dissection of the regional and mediastinal lymph nodes (pNX-category), or in patients scheduled to undergo stereotactic ablative radiotherapy (SABR). However, adjuvant chemotherapy can also be discussed in these patients. Furthermore, improving in the core needle technique, which could provide more tissue to be examined, might be useful in enhancing the detection rate of L-positive patients preoperatively.

The aim of this study was to evaluate the risk factors of lymph node metastasis in operable NSCLSs, and, consequently, to evaluate oncologically adequate treatment strategies. Limitations of this study are retrospective analyses and non-inclusion of potentially prognostic parameters such as CEA, which were not available.

## Conclusions

NSCLC-patients with larger size tumors and central tumor localization require a precise preoperative and intraoperative assessment of their lymph node status, as these patients have an increased risk of lymph node metastasis. L1-status is a risk factor for lymph node metastasis in NSCLCs, irrespective of tumor size. In a situation with no sufficient postoperative lymph status (pNX), this risk factor should be considered when determining the postoperative (adjuvant) treatment. However, further studies are required to investigate the effects of adjuvant chemotherapy in cases with L1-status.

## Abbreviations

CEA: Serum carcinomembryonic antigen
EBUS: Endobronchial Ultrasound
IASLC: International Association for Study of Lung Cancer
MD-ATS map: Mountain, Dresler –American Thoracic Society map
MLNS: Mediastinal lymph node sampling
NSCLC: Non-small cell lung cancer
SABR: Stereotactic ablative radiotherapy
SCC: Suqamous cell carcinomas
SND: Systematic node dissection
TBNA: Transbronchial Needle Aspiration
VAM: Video-assisted mediastinoscopy
VAMLA: Video-assisted mediastinal lymphadenectomy
VATS: Video-assisted thoracoscopy
