# Correction to: Predictive risk factors for lymph node metastasis in patients with resected non-small cell lung cancer: a case control study

**DOI:** 10.1186/s13019-019-0850-x

**Published:** 2019-02-05

**Authors:** Yusef Moulla, Tanja Gradistanac, Christian Wittekind, Uwe Eichfeld, Ines Gockel, Arne Dietrich

**Affiliations:** 1 Department of Visceral, Transplant, Thoracic and Vascular Surgery, University Hospital of Leipzig, Liebigstraße 20, 04103 Leipzig, Germany; 2 Institute of Pathology, University Hospital of Leipzig, Liebigstraße 20, 04103 Leipzig, Germany

**Correction to: J Cardiothorac Surg**

**https://doi.org/10.1186/s13019-019-0831-0**

The original article [1] contains slight errors whereby several terms in the first column of Tables 1, 2, and 3 have an erroneous ‘p’ preceding them. The correct presentation of these tables can be viewed ahead.

**Table 1:** Main characteristics of the patients, pTNM-Classification 2017, 8th Edition

Characteristics	Number of Patients (n)	Percentage
Tumor-localization		
central	53	26.2%
peripheral	149	73.8%
Histology		
adenocarcinoma	117	57.4%
SCC^a^	75	36.8%
others	12	5.9%
Grading		
In situ	2	1%
G1	25	12.4%
G2	95	47.0%
G3	68	33.3%
G4	12	5.9%
pT-category		
pTis	2	1%
pT1	90	45.1%
pT1a	10	4.9%
pT1b	37	18.1%
pT1c	43	21.1%
pT2	44	26.5
pT2a	39	19.1%
pT2b	15	7.4%
pT3	35	17.2%
pT4	23	11.3
pN-category		
pN0	126	61.8%
pN1	33	16.2%
pN2	45	22%
pM-category		
pM0	193	94.5%
pM1a	5	2.5%
pM1b	5	2.5%
pM1c	1	0.5%
L-Status		
L0	57	28.1%
L1	146	71.9%
V-Status		
V0	165	81.3%
V1	38	18.7%

^a^*SCC* Squamous cell carcinoma

**Table 2:** Univariate Analysis of positive pulmonary and mediastinal lymph nodes (pN1+, pN2+)

Variables	pN1- (%)	pN1+ (%)	*P*-Value	pN2- (%)	pN2+ (%)	*P*-Value
Sex			0.148			0.627
male	90 (63.4)	52 (36.6)		112 (78.9)	30 (21.1)	
female	46 (74.2)	16 (25.8)		47 (75.8)	15 (24.2)	
Age			0.298			0.237
≤ 68 years	68 (63)	40 (37)		88 (81.5)	20 (81.5)	
> 68 years	68 (70.8)	28 (29.2)		71 (74.0)	25 (21.2)	
Tumor site			0.067			0.783
right	90 (71.4)	36 (28.6)		99 (78.6)	27 (21.4)	
left	46 (59)	32 (41)		60 (76.9)	18 (23.1)	
Tumor localization			< 0.001			0.02
central	25 (47.2)	28 (52.8)		36 (66.7)	18 (33.3)	
peripheral	111 (74.5)	38 (25.5)		122 (81.9)	27 (18.1)	
Tumor size			0.011			0.042
≤ 3 cm	77 (75.5)	25 (24.5)		86 (84.3)	16 (15.7)	
> 3 cm	59 (57.8)	43 (42.8)		73 (71.6)	29 (28.4)	
Lobe distribution			0.918			0.385
UL^a^	85 (66.4)	43 (33.6)		97 (75.8)	31 (24.2)	
Non-UL	51 (67.1)	25 (32.9)		62 (81.6)	14 (18.4)	
pT-category			0.015			0.081
pT1	70 (77.8)	20 (22.2)		77 (85.6)	13 (14.4)	
pT2	33 (61.1)	21 (38.9)		40 (74.1)	14 (25.9)	
pT3	18 (51.4)	17 (48.6)		23 (65.7)	12 (34.3)	
pT4	13 (56.5)	10 (43.5)		17 (73.9)	6 (26.1)	
R-classification			0.017			0.088
R0	130 (68.8)	59 (31.2)		150 (79.4)	39 (20.6)	
R1–2	5 (35.7)	9 (64.3)		8 (57.1)	6 (42.9)	
L-status			< 0.001			< 0.001
L0	55 (96.5)	2 (3.5)		56 (98.2)	1 (1.8)	
L1	80 (54.8)	66 (45.2)		102 (69.9)	44 (30.1)	
V-status			0.006			0.004
V0	117 (70.9)	48 (29.1)		135 (81.1)	30 (18.2)	
V	18 (47.4)	20 (52.6)		23 (60.5)	15 (39.5)	
Pn-Status			0.088			0.34
Pn0	131 (67.9)	62 (32.1)		149 (77.2)	44 (22.8)	
Pn1	4 (40.0)	6 (60.0)		9 (90.0)	1 (10.0)	
Histological tumor type			0.214			0.646
Adeno-Ca.^b^	84 (71.8)	33 (28.2)		94 (80.3)	23 (19.7)	
				56 (74.4)	19 (25.6)	
SCC^c^	45 (60.0)	30 (40.0)		9 (75.0)	3 (25.0)	
others	7 (58.3)	5 (41.7)				
Grading			0.016			0.001
G1	25 (89.3)	3 (10.7)		27 (96.4)	1 (3.6)	
G2	64 (67.4)	31 (32.6)		79 (83.2)	16 (16.8)	
G3	39 (57.4)	29 (42.6)		43 (63.2)	25 (36.8)	
G4	7 (58.3)	5 (41.7)		9 (75.0)	3 (25.0)	

^a^*UL* Upper lobe, ^b^*Adeno-Ca.* Adenocarcinoma, ^c^*SCC* Squamous cell carcinoma

**Table 3:** Univariate Analysis of positive lymph nodes (pN0, pN+)

Variables	pN0 (%)	pN+ (%)	*P*-Value
Sex			0.397
male	85 (59.9)	57 (40.1)	
female	41 (66.1)	21 (33.9)	
Age			0.314
≤ 68 years	63 (58.3)	45 (41.7)	
> 68 years	63 (65.6)	33 (34.4)	
Tumor site			0.067
Right	84 (66.7)	42 (33.3)	
Left	42 (53.8)	36 (46.2)	
Tumor localization			< 0.001
central	22 (41.5)	31 (58.5)	
peripheral	104 (69.8)	45 (30.2)	
Tumor size			< 0.001
≤ 3 cm	76 (74.5)	26 (25.5)	
> 3 cm	50 (49)	52 (51)	
Lobe distribution			0.834
UL ^a^	78 (60.9)	50 (39.1)	
Non-UL	40 (62.5)	24 (37.5)	
Number of dissected LN ^b^			0.005
pT-category			0.001
pT1	69 (76.7)	21 (23.3)	
pT2	27 (50.9)	26 (49.1)	
pT3	17 (47.2)	19 (52.8)	
pT4	11 (47.8)	12 (52.2)	
R-classification			0.048
R0	120 (96.0)	5 (4.0)	
R1–2	69 (88.5)	9 (11.5)	
L-status			< 0.001
L0	54 (94.7)	3 (5.3)	
L1	71 (48.6)	75 (51.4)	
V-status			0.001
V0	111 (67.3)	54 (32.7)	
V1	14 (36.8)	24 (63.2)	
Pn-status			0.188
Pn0	121 (96.8)	4 (3.2)	
Pn1	72 (92.3)	6 (7.7)	
Histological tumor type			0.141
Adeno-Ca.^b^	79 (67.5)	38 (32.5)	
SCC^c^	41 (54.7)	34 (45.3)	
others	6 (50.0)	6 (50.0)	
Grading			0.005
G1	22 (88.0)	3 (12.0)	
G2	60 (63.2)	35 (36.8)	
G3	34 (50.0)	34 (50.0)	
G4	6 (50.0)	6 (50.0)	

^a^*UL* Upper lobe, ^b^*Adeno-Ca.* Adenocarcinoma, ^c^*SCC* Squamous cell carcinoma
